# The impact of the COVID-19 pandemic on thyroid nodular disease: a retrospective study in a single center in the western part of Romania

**DOI:** 10.3389/fendo.2023.1221795

**Published:** 2023-07-11

**Authors:** Oana Popa, Robert Alexandru Barna, Andreea Borlea, Marioara Cornianu, Alis Dema, Dana Stoian

**Affiliations:** ^1^ Endocrinology Department, Centre of Molecular Research in Nephrology and Vascular Disease, University of Medicine and Pharmacy “Victor Babes”, Timisoara, Romania; ^2^ Discipline of Morphopathology, Department of Microscopic Morphology, ANAPATMOL Research Centre, University of Medicine and Pharmacy “Victor Babes”, Timisoara, Romania

**Keywords:** COVID-19, nodular thyroid disease, thyroid cancer, healthcare system, surgery

## Abstract

**Introduction:**

The COVID-19 pandemic had a significant impact on the healthcare system, leading to a prioritization of hospital admissions in many countries. Romania was no exception, and it had to restrict patient access to medical services in hospitals with chronic diseases and oncological pathology, including thyroid cancer. This study aimed to compare the clinical and pathological factors of patients with nodular thyroid disease diagnosed and surgically treated during the two years before and after the COVID-19 pandemic, in a single medical institution.

**Methods:**

The retrospective study included 1505 patients who were diagnosed and operated on for nodular thyroid disease between January 2018 and December 2021. The patients were divided into two groups: the “PRECOVID” group (January 2018 to February 2020), and the “POSTCOVID” group (March 2020 to December 2021). The analyzed parameters included patients’ gender, age, preoperative diagnosis, type of surgical intervention, and pathological diagnosis.

**Results:**

A significant decrease was observed in the number of surgeries performed for thyroid nodular disease during the COVID-19 pandemic period (450 versus 1055 cases, p<0.00001). There was a significant decrease in the number of surgical reinterventions (0.9% in the POSTCOVID group versus 2.9% in the PRECOVID group, p=0.01) and a significant increase in the number of total thyroidectomies (84.9% in the POSTCOVID group versus 80.1% in the PRECOVID group, p=0.02). We also observed a higher incidence of malignant/borderline tumors in the POSTCOVID group compared to the PRECOVID group (p=0.04) and a significantly higher frequency of aggressive forms of thyroid cancer in the POSTCOVID group (p=0.0006).

**Discussion:**

The COVID-19 pandemic had a significant impact on the surgical management of nodular thyroid disease, resulting in a decrease in surgeries and a change in the type of surgical interventions performed. The higher incidence of malignant/borderline tumors diagnosed during the pandemic highlights the importance of timely diagnosis and treatment of thyroid nodules to prevent cancer progression.

## 1 Introduction

The healthcare system in our country, similar to Europe, starting from March 2020 (1), had to adjust and prioritize hospital admissions to cope with the overwhelming wave of COVID-19-positive cases. The state of emergency and lockdown, active between March 15, 2020, and May 15, 2020 (2), restricted the access of patients with chronic pathology to medical services in hospitals, a situation often encountered worldwide and mentioned in numerous studies (3–8). Within a relatively short period, specific protocols and guidelines were developed, recommending the rescheduling of certain surgical interventions, including those in the oncology field (9–11).

Despite these inconveniences, medical teams consisting of endocrinologists, surgeons, radiologists, and pathologists at our institution, the largest hospital in Western Romania, evaluated patients presenting with nodular thyroid disease and selected patients that needed immediate medical attention. Each case and therapeutic decision was carefully analyzed from the perspective of the benefit-risk balance.

Thyroid carcinoma represents the most frequent endocrine malignant tumor worldwide. Recent studies state that the incidence has tripled in the last 20 years (12). The most recent World Health Organization (WHO) report estimates that in 2020, 449,000 new cases were diagnosed in female and 137,000 in male patients (13). Taking into consideration that thyroid cancer has a much more favorable outcome compared to other types of malignant tumors, recent guidelines mention that the diagnosis and surgical intervention could be postponed during the COVID-19 pandemic without detrimental effects on the patient’s survival and prognosis (14–16).

Our study aims to evaluate the clinical and pathological factors of patients with nodular thyroid disease by comparing the cases that were diagnosed and surgically treated during the 2 years before and after the COVID-19 pandemic in a single medical institution.

## 2 Materials and methods

We have conducted a retrospective study comprising all cases that were diagnosed with nodular thyroid disease and surgically treated in the three surgical departments of Timisoara County Hospital “Pius Brinzeu”, between January 2018 and December 2021. The patients were divided into two groups: the pre-pandemic group from January 2018 to February 2020 called the “PRECOVID” group (1055 cases) and the post-pandemic group from March 2020 to December 2021 called the “POSTCOVID” group (450 cases). The working protocol was approved by the hospital’s ethics committee and the patients signed informed consent.

All types of thyroid surgeries were included in the study, including total thyroidectomies, unilateral lobectomies, and reinterventions for completion of thyroidectomy after the diagnosis of malignancy or recurrence of suspicious nodules following initial lobectomy. All cases included in the study were examined by an experienced pathologist on HE-stained sections. Immunohistochemistry was performed in challenging cases.

The analyzed parameters were the patient’s gender, age, preoperative diagnosis, type of surgical intervention, and pathological diagnosis. For the systematization of the results and statistical analysis, non-tumor lesions were classified as follows: cases of nodular goiter (NG), nodular goiter evaluated through fine needle aspiration cytology (NG with FNAC), compressive nodular goiters (NG-compressive), hyperthyroid goiter (HTG), Graves’ disease (GD), and chronic autoimmune thyroiditis with thyroid nodules (CAT ± nodules). We have included borderline thyroid tumors/lesions, due to their uncertain malignant potential, in the malignant tumor category, alongside papillary thyroid carcinoma (PTC), micropapillary thyroid carcinoma (micro-PTC), follicular thyroid carcinoma (FTC), medullary thyroid carcinoma (MTC), anaplastic thyroid carcinoma (ATC), and other rare types of tumors. The borderline thyroid tumors/lesions included in the study are noninvasive follicular thyroid neoplasm with papillary-like nuclear features (NIFT-P), well-differentiated thyroid tumors of uncertain malignant potential (WDT-UMP), and follicular tumors of uncertain malignant potential (FT-UMP).

Statistical analyses were conducted using MedCalc version 20.218 (MedCalc Software Ltd, Belgium). Quantitative variables were presented as mean ± standard deviation (SD) and qualitative data were reported in frequency and percentages. To compare the proportions between two independent samples, we used the two-sample proportion test, and for testing differences between two population means, we used the independent samples t-test. To assess the association between categorical variables, the chi-square test was used. All statistical tests were two-tailed unless otherwise specified. To establish statistical significance for the test results, we employed a predetermined threshold of p-value less than 0.05.

## 3 Results

The retrospective study included 1505 patients diagnosed and operated for nodular thyroid disease between January 2018 and December 2021, of which 36 patients underwent reintervention. Postoperatively, 992 non-tumor lesions (65.9%) and 513 malignant/borderline tumors (34.1%) were diagnosed.

The results of our study showed that there were significantly fewer surgeries performed for thyroid nodular disease during the time frame of 2020-2021, in comparison with the earlier years (450 cases versus 1055 cases, p<0.00001; **Table 1**). The distribution of the cases in the two groups was similar in terms of the patient’s gender and median age. We noticed a significant decrease in the number of surgical reinterventions in the post-COVID period (4 cases, 0.9%) compared to the PRECOVID group (32 cases, 2,9%: p=0.01) and an increase in the number of total thyroidectomies (382 cases, 84.9%) in comparison to the PRECOVID group (870 cases, 80,1%; p=0.02). We observed that significantly more cases of malignant/borderline tumors were diagnosed during the period of 2020-2021 compared to non-tumor lesions in the PRECOVID group (p=0.04).

**Table 1:** Clinical and pathological factors in the PRECOVID and POSTCOVID study groups.

Clinical and morphological parameter	PRECOVID No. of cases (%)	POSTCOVID No. of cases (%)	p-value	p-value for each parameter
**Number of cases**	1055	450	p<0.00001	p<0.00001
**Men**	105 (9,95%)	52 (11,6%)	p=0.36	p=0.35
**Women**	950 (90,05%)	398 (88,4%)		p=0.35
**Median age**	53,6 ± 18	54,2 ± 11	p=0.51	p=0.51
Type of surgical intervention				
- **Total thyroidectomy**	870 (80,1%)	382 (84,9%)	p=0.02	p=0.25
- **Lobectomy**	185 (17%)	64 (14,2%)		p=0.11
- **Reintervention**	32 (2,9%)	4 (0,9%)		p=0.01
Presurgical diagnosis				
- **NG**	708 (65,1%)	301 (66,9%)	p<0.0001	p=0.94
- **NG with FNAC**	130 (12%)	66 (14,6%)		p=0.22
- **NG-compressive**	54 (5%)	40 (8,9%)		p=0.006
- **HTG**	43 (3.9%)	13 (2,9%)		p=0.27
- **GD**	65 (6%)	21 (4,7%)		p=0.25
- **CAT ± nodules**	55 (5,1%)	5 (1,1%)		p=0.0002
- **Reintervention**	32 (2,9%)	4 (0,9%)		p=0.01
**Non-tumor lesions**	715 (67,8%)	277 (62,1%)	p=0.04	p=0.02
**Malignant/borderline tumor**	340 (32,2%)	169 (37,9%)		p=0.05

NG, nodular goiter; FNAC, nodular goiter evaluated through fine needle aspiration cytology; NG-compressive, compressive nodular goiters; HTG, hyperthyroid goiter; GD, Graves’ disease; CAT, chronic autoimmune thyroiditis with thyroid nodules; CAT ± nodules, chronic autoimmune thyroiditis with thyroid nodules.

Postoperatively, diagnosed non-tumor lesions presented a relatively similar distribution between the two study groups (**Table 2**). By analyzing the cases of malignant and borderline tumors, we noticed a slight increase in the number of cases diagnosed in the POSTCOVID period for the age groups of 31-50 years (13.7% versus 11.3%) and 51-65 years (14.1% versus 13.6%), but the number of cases increased significantly for patients over 65 years old (9.2% versus 6.5%), both for women (22.4% versus 19.6%), but especially for men (36.4% versus 23.6%), although the statistical analysis did not show a significant correlation.

**Table 2:** Distribution of tumoral and non-tumoral lesions among the two study groups.

Pathological diagnostic	PRECOVID No. of cases (%)	POSTCOVID No. of cases (%)	p-value	p-value for each parameter
**Non-tumoral lesions**	715 (67,8%)	277 (62,1%)		p=0.02
** NG**	465 (44,1%)	192 (43%)	p=0.25	p=0.2
** CAT**	153 (14,5%)	44 (9,9%)		p=0.05
** GD**	57 (5,4%)	22 (4,9%)		p=0.98
** HTG**	40 (3,8%)	19 (4,3%)		p=0.45
**Malignant/borderline tumor**	340 (32,2%)	169 (37,9%)		p=0.05
** < 30 years**	9 (0,85%)	4 (0,9%)	p=0.68	p=0.85
** 31-50 years**	119 (11,3%)	61 (13,7%)		p=0.81
** 51-65 years**	143 (13,6%)	63 (14,1%)		p=0.3
** >65 years**	69 (6,5%)	41 (9,2%)		p=0.31
**Women**	285 (27%)	147 (33%)		p=0.03
** < 30 years**	8 (2,8%)	3 (2,1%)	p=0.88	p=0.63
** 31-50 years**	103 (36,2%)	53 (36,1%)		p=0.98
** 51-65 years**	118 (41,4%)	58 (39,4%)		p=0.7
** >65 years**	56 (19,6%)	33 (22,4%)		p=0.5
**Men**	55 (5,2%)	22 (4,9%)		p=0.8
** < 30 years**	1 (1,8%)	1 (4,5%)	p=0.29	p=0.5
** 31-50 years**	16 (29,1%)	8 (36,4%)		p=0.54
** 51-65 years**	25 (45,5%)	5 (22,7%)		p=0.06
** >65 years**	13 (23,6%)	8 (36,4%)		p=0.26
**PTC**	135 (39,7%)	48 (28,4%)	p=0.001	p=0.01
**Micro-PTC**	140 (41,2%)	77 (45,6%)		p=0.35
**Borderline tumors**	39 (11,5%)	14 (8,3%)		p=0.27
**Other malignant tumors**	26 (7,6%)	30 (17,7%)		p=0.0006

NG, nodular goiter; HTG, hyperthyroid goiter; GD, Graves’ disease; CAT, chronic autoimmune thyroiditis with thyroid nodules.

**Figure 1:**
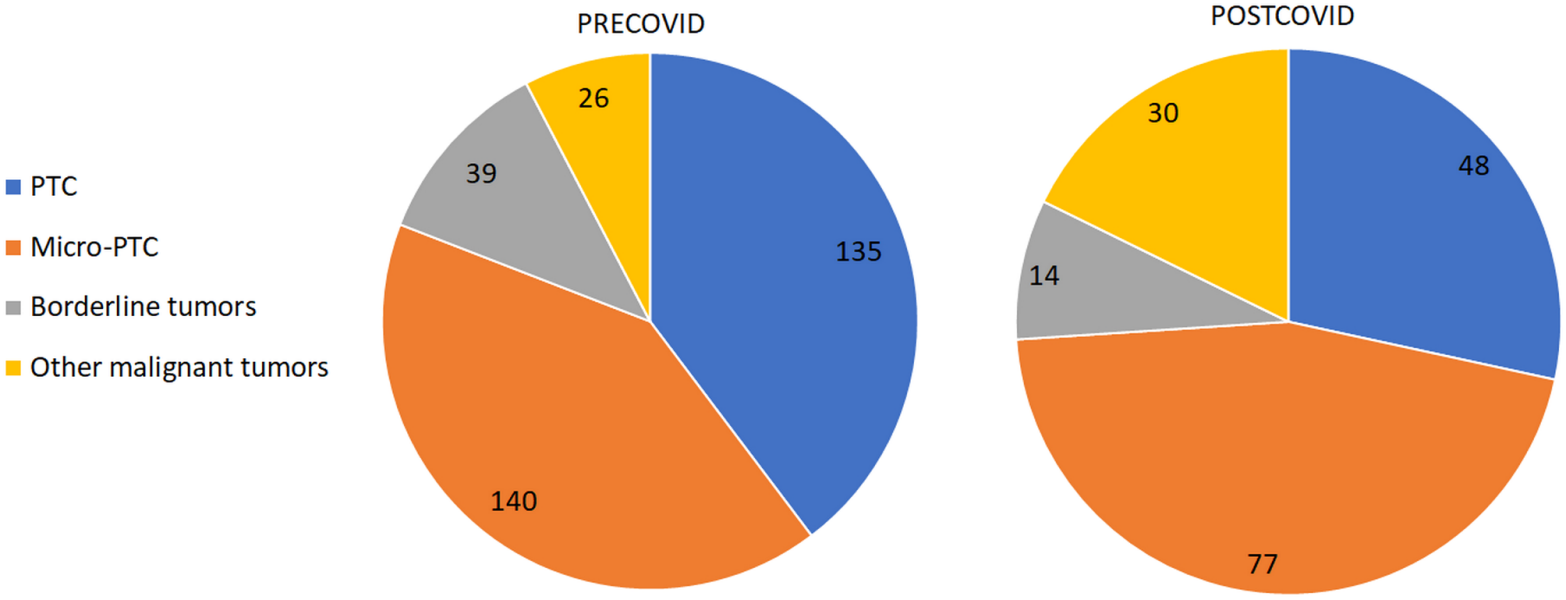
The comparison of tumor types between PRECOVID and POSTCOVID cases.

The comparative study of malignant and borderline tumor cases (**Figure 1**) highlighted a statistically significant decrease in the number of PTC diagnosed and surgically treated during the post-COVID period (28.4% versus 39.7%, p=0.01), with a slight increase in the number of cases of micro-PTC (45.6% versus 41.2%) and a statistically significant increase in the number of patients with other malignant tumors (17.7% versus 7.6%, p=0.0006). During the time frame of 2020-2021, we identified 14 FTC, 9 MTC, and 5 ATC cases, as well as one case of diffuse large B-cell lymphoma and one case of thyroid metastasis from lung adenocarcinoma.

The comparative analysis of the study groups regarding the concordance between presurgical diagnosis and pathological diagnosis is presented in **Table 3**. In the POSTCOVID group, we observed more cancers and borderline lesions diagnosed in patients with NG (38.8%) compared to the PRECOVID group (27,5%). Significant increases in malignant lesions in the period of 2020-2021 were observed in patients diagnosed preoperatively with HTG (15.4% versus 9.3%), CAT ± nodules (40% versus 30.9%), and GD (33.3% versus 29,2%).

**Table 3:** The correspondence between presurgical diagnosis and surgical pathology reports.

Clinical/ Pathological diagnosis	PRECOVID						POSTCOVID					
	No. of cases	NG	CAT	GD	HTG	M/B tumor	No. of cases	NG	CAT	GD	HTG	M/B tumor
NG	708	387	114	3	9	195 (27,5%)	407	172	40	5	16	158 (38,8%)
NG with FNAC	130	49	–	–	–	81 (62,3%)	66	29	5	1	–	31 (47%)
NG-compressive	54	26	4	–	–	24 (44,4%)	40	16	5	1	4	14 (35%)
HTG	43	–	2	8	29	4 (9,3%)	13	3	1	4	3	2 (15,4%)
CAT ± nodules	55	3	33	1	1	17 (30.9%)	5	–	3	–	–	2 (40%)
GD	65	–	–	45	1	19 (29,2%)	21	1	–	13	–	7 (33,3%)

NG, nodular goiter; NG with FNAC, nodular goiter evaluated through fine needle aspiration cytology; NG-compressive, compressive nodular goiters; HTG, hyperthyroid goiter; GD, Graves’ disease; CAT ± nodules, chronic autoimmune thyroiditis with thyroid nodules.

## 4 Discussion

The WHO declared COVID-19 a global pandemic on the 11th of March 2020 (1). Social distancing measures and the state of lockdown have profoundly affected the healthcare system in almost all parts of the world (17). As the medical team focused primarily on COVID-positive patients, newly implemented oncology guidelines recommended short-term deferral of certain procedures and surgeries, including cases of aggressive cancers such as gastric, pancreatic, genitourinary, or melanoma (14, 18). Some studies have shown that 38.7% to 59% of cancer patients were advised to delay treatment (12, 19, 20).

Thyroid cancer is the most common endocrine malignancy, with surgical intervention being the first-line therapy. Although the mortality rate is less than 1 per 100,000, delaying treatment can lead to the local extension of neoplastic disease, requiring extensive surgical interventions and sometimes radioactive iodine treatment, with the risk of complications such as hypoparathyroidism, laryngeal recurrent nerve damage, and aesthetic complications due to poor scarring (14, 21). Some studies have shown that extensive surgical interventions and radioactive treatment do not influence the risk of recurrence and survival of patients with micro-PTC (22, 23). The recent guidelines of the American Thyroid Association recommend lobectomy for patients with PTC under 4 cm, without clinical evidence of local extension or lymph node metastasis (22).

The COVID pandemic has affected the diagnosis and surgical treatment of many oncological pathologies, including thyroid nodular disease and, consequently, thyroid cancer (14, 15, 24). Numerous guidelines developed by reputable societies in thyroid pathology recommended delaying surgical interventions for thyroid cancer, except for those cases that endanger patients’ lives (15, 16, 25). Studies by Medas et al. and Bakkar et al. have shown that there are no significant differences in the progression of patients with thyroid cancer before and after the COVID pandemic (26, 27). On the other hand, a study in China reported an increased incidence of severe prognostic factors, such as extrathyroidal extension, multiple tumors, and lymph node metastasis (12). In a study that analyzed the survival of patients with thyroid disease, 87.9% of clinicians expressed concern about delayed treatment during the pandemic (28).

We included in our study all patients that were diagnosed and operated on for thyroid nodular disease in a single medical institution, the largest hospital in western Romania, over 4 years (1505 patients): 2 years before and 2 years after the declaration of the COVID pandemic. We can report that the COVID pandemic has significantly affected the diagnosis and treatment of patients with nodular thyroid disease. Between 2020 and 2021, 450 patients underwent thyroid surgery, significantly fewer patients than in the pre-pandemic period (1055 patients, p<0.00001). The two groups of patients were similar in terms of patient’s gender and age. Our results suggest that in the post-pandemic period, surgeons preferred total thyroidectomy as the first therapeutic option (POSTCOVID 84.9% versus PRECOVID 80.1%, p=0.02) and limited surgical reinterventions (POSTCOVID 0.9% versus PRECOVID 2,9%, p=0.01). Another important result of our study indicated an increase in the number of malignant tumors and borderline thyroid lesions in the post-pandemic years (37.9% versus 32.2%; p=0.04). Non-tumoral lesions showed a relatively similar distribution between the two study groups.

Thyroid cancers and borderline thyroid tumors were diagnosed more frequently in the POSTCOVID group in all age groups, especially in patients over 65 years old (9.2% versus 6.5%) and in men (36.4% versus 23.6%). Our results suggest that elderly patients had reduced access to healthcare due to the greater risk of infection and more severe complications associated with COVID-19.

One of the most important results of our study is the significantly higher frequency of aggressive pathological tumor types in the POSTCOVID group (17.7%) compared to the PRECOVID group (7.6%; p=0.0006). A plausible explanation could be the delay in diagnosis and surgical interventions in the immediate period following the declaration of the COVID pandemic. Certain types of thyroid cancer, such as FTC, MTC, or ATC, are characterized by rapid growth, extrathyroidal extension, and early lymph node metastases (12). In the case of these malignant tumors, delaying surgical intervention, even for a few months, is not desirable or advisable.

Although significantly fewer patients with NG were operated on during the period of 2020-2021, the number of cases diagnosed with malignant tumors and borderline lesions was higher (POSTCOVID - 38.8% versus PRECOVID – 27,5%). Significant increases in malignant lesions in the period of 2020-2021 were also observed in patients diagnosed before surgery with HTG (15.4% versus 9.3%), CAT ± nodules (40% versus 30.9%), and GD (33.3% versus 29,2%)

Presurgical ultrasound (US) evaluation and risk stratification show excellent accuracy for conventional US, especially when combined with elastography assessment. Presurgical evaluation is needed to reduce the unnecessary number of surgical interventions (21, 29, 30). In our study, the high frequency of total thyroidectomies is justified by the increased number of diagnosed thyroid cancers, especially in the POSTCOVID group.

One of the limitations of our study is that the analysis was carried out in a single medical center. Multicenter research is needed to include a larger number of cases to be able to draw an accurate conclusion about the effects of the pandemic on patients with nodular thyroid disease. Additionally, not all patients suspected of thyroid cancer underwent fine-needle aspiration (FNA), which could lead to the underdiagnosis of aggressive forms of cancer.

## 5 Conclusion

Our results allow us to state that the COVID pandemic has significantly affected the diagnosis and treatment of patients with thyroid nodular disease. After the onset of the COVID pandemic, the number of patients diagnosed and operated on for nodular thyroid disease decreased dramatically. However, the number of thyroid cancer cases did significantly increase, especially for aggressive tumors such as FTC, CMT, and ATC, suggesting better presurgical evaluations of the cases. Between 2020 and 2021, the number of cases of thyroid cancer increased significantly for all age groups, particularly for elderly patients (aged 65 and older).

Under the exceptional conditions imposed by the pandemic, surgeons preferred total thyroidectomies over unilateral lobectomies or reinterventions.

Our study results suggest that delaying diagnosis and treatment during the COVID pandemic, even by a few months, is not the best option for patients with aggressive forms of thyroid cancer. However, multicenter studies are necessary to include a large case series to support our current observations.

## Data availability statement

The raw data supporting the conclusions of this article will be made available by the authors, without undue reservation.

## Ethics statement

The working protocol was approved by the hospital’s ethics committee and the patients signed informed consent.

## Author contributions

OP was involved in the conceptualization, methodology, validation, supervision, and reviewing and editing of the manuscript; RB performed statistical analysis and visualization. AB was involved in the investigation and writing the original draft; MC and AD were involved in the investigation, resources, and writing the original manuscript; DS was involved in the supervision, validation, reviewing, and editing of the manuscript. All authors contributed to the article and approved the submitted version.
